# Leaning Into the Bite: The piRNA Pathway as an Exemplar for the Genetic Engineering Need in Mosquitoes

**DOI:** 10.3389/fcimb.2020.614342

**Published:** 2021-01-14

**Authors:** Vanessa M. Macias, Umberto Palatini, Mariangela Bonizzoni, Jason L. Rasgon

**Affiliations:** ^1^Department of Entomology, The Pennsylvania State University, University Park, PA, United States; ^2^Department of Biology and Biotechnology, University of Pavia, Pavia, Italy; ^3^Huck Institutes of the Life Sciences, The Pennsylvania State University, University Park, PA, United States; ^4^Center for Infectious Disease Dynamics, The Pennsylvania State University, University Park, PA, United States

**Keywords:** vector-borne disease, Piwi-interacting RNAs, mosquito basic biology, host-pathogen interactions, genetic engineering technologies

## Abstract

The piRNA pathway is a specialized small RNA interference that in mosquitoes is mechanistically distant from analogous biology in the *Drosophila* model. Current genetic engineering methods, such as targeted genome manipulation, have a high potential to tease out the functional complexity of this intricate molecular pathway. However, progress in utilizing these methods in arthropod vectors has been geared mostly toward the development of new vector control strategies rather than to study cellular functions. Herein we propose that genetic engineering methods will be essential to uncover the full functionality of PIWI/piRNA biology in mosquitoes and that extending the applications of genetic engineering on other aspects of mosquito biology will grant access to a much larger pool of knowledge in disease vectors that is just out of reach. We discuss motivations for and impediments to expanding the utility of genetic engineering to study the underlying biology and disease transmission and describe specific areas where efforts can be placed to achieve the full potential for genetic engineering in basic biology in mosquito vectors. Such efforts will generate a refreshed intellectual source of novel approaches to disease control and strong support for the effective use of approaches currently in development.

## Introduction

**Figure 1:**
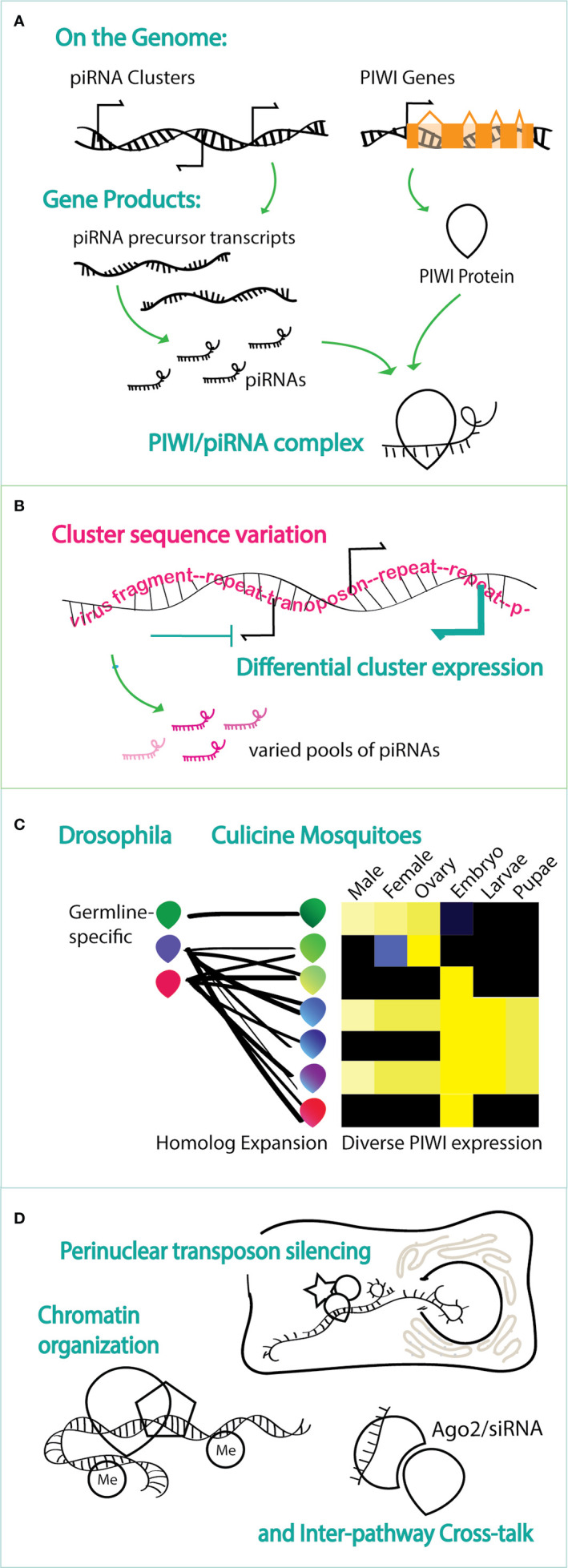
Schematics of endogenous piRNA pathway activity, highlighting the molecular phenomena that contribute to functional complexity. **(A)** piRNAs, from piRNA clusters, and PIWI proteins, encoded by PIWI genes, are the basic functional unit whereby a target is recognized by piRNA sequence. **(B)** piRNAs present in a given context vary and are dictated by the sequences present in piRNA clusters (as depicted in pink) and by whether and how much a cluster is expressed in that context (depicted in blue). **(C)** Increased functional complexity in Culicine mosquitoes is added by the expansion of PIWI proteins in mosquitoes with respect to *Drosophila* and by the varied expression across tissues and stages in Culicine mosquitoes compared to primarily germ-line specific expression of PIWIs in Drosophila. Heat map schema is based on data from Akbari et al. (2013). **(D)** Protein binding partners add a level functional complexity, including identified roles of the PIWI/piRNAs complex in with perinuclear proteins for transcript silencing, interaction with protein complexes that add marks to the chromatin, association with the siRNA pathway and, in mosquitoes, with viruses.

The piRNA pathway (PIWI-interacting RNA pathway) is a fascinating biological system that allows an organism to identify parasitic nucleic acids in its cells and create a heritable, genetic memory of these exogenous sequences (**Figures 1A–D**, Siomi et al., 2011). Arrays of degraded or partial sequences from transposons and viruses are clustered into discrete genomic loci termed piRNA clusters. Very short ribonucleic acids (>24 nt in length), now called piRNAs are encoded from piRNA clusters (Aravin et al., 2006; Vagin et al., 2006; Brennecke et al., 2007). Mature piRNAs, in concert with PIWI (P-element induced wimpy testis) proteins target homologous RNA sequences for destruction (Lin and Spradling, 1997). The activity against a specific target can be amplified by ping-pong amplification, where-by additional piRNAs are generated by interaction with the target RNA and PIWI paralogs (Brennecke et al., 2007; Czech and Hannon, 2016). Complexity of function within an organism is added by protein binding partners that interact with the PIWI/piRNA complex and direct or influence its localization and activity (Brower-Toland et al., 2007; Czech and Hannon, 2011; Arkov, 2018). Many specific features of this system are unknown. For example, piRNA clusters harbor sequences that represent a history of exposure to these invasive agents that is different among species and perhaps across populations of the same species, but we have little understanding of how foreign sequences are integrated into piRNA clusters (Blair et al., 2020).

The specialization of piRNA pathways among species has captivated the minds of scientists who study diverse organisms with questions about their activity in somatic tissues and roles such as in stem cell maintenance, genome architecture and integrity, behavior, evolution and cancer (Gibson et al., 2015; Lewis et al., 2018; Waldron et al., 2018; Liu et al., 2019; Ozata et al., 2019). Although mosquitoes share hallmark piRNA pathway features with *Drosophila*, such as phased biogenesis and ping-pong signatures resulting in 1U and 10A trends on piRNAs (Gainetdinov et al., 2018), specialized features of piRNA pathway function is evident in mosquitoes (Gamez et al., 2020). A prominent difference is the antiviral activity of the piRNA pathway observed in mosquitoes, but not *Drosophila* (Petit et al., 2016). piRNAs that may regulate virus replications are generated both from the virus genome (Hess et al., 2011; Morazzani et al., 2012; Vodovar et al., 2012; Léger et al., 2013; Miesen et al., 2015; Miesen et al., 2016; Göertz et al., 2019) and from Endogenous Virus Elements (EVEs) in the mosquito genome, fragments of viruses often integrated into piRNA clusters (Lequime and Lambrechts, 2017; Palatini et al., 2017; Suzuki et al., 2017; Whitfield et al., 2017; ter Horst et al., 2018; Aguiar et al., 2020; Blair et al., 2020; Suzuki et al., 2020). Further specialization in mosquitoes is evidenced in both *Aedes* and *Anopheles* species: 1) *PIWI* genes and other piRNA pathway genes have expanded and diverged in *Aedes* mosquitoes (Bernhardt et al., 2012; Miesen et al., 2015; Marconcini et al., 2019; Ma et al., 2020). 2) Cluster content, number and genomic distribution is diverged in both genera [(George et al., 2015; Crava et al., 2020; Palatini et al., 2020). 3) A strong activity of PIWIs and piRNAs in the soma and 4] multiple distinct roles in gene expression are evident in both genera (Belda et al., 2019; Halbach et al., 2020). As such, the piRNA pathway represents an enigmatic biological system in mosquitoes, warranting a more aggressive study of the system in mosquito species.

## The piRNA Pathway Exemplifies Basic Biology in Mosquitoes That is Important, But Enigmatic

Knowledge of the basic interactions between pathogens and hosts serves as basis for the development of strategies to interrupt those interactions. From what is already understood about the piRNA pathway, several possible applications to disease control can be imagined. The piRNA pathway genetically encodes targeted destruction of RNA. This brings forward several questions: is it possible that we could engineer the pathway to recognize and repress viral RNA, preventing transmission to humans? Knowing that this biology targets foreign and integrated mobile DNA elements, is it possible that genetically modified mosquitoes could also recognize transgenes, such as those being developed and deployed for public health efforts to mitigate mosquito-borne diseases? If the answer to either of these questions is yes, a myriad of additional mechanistic questions will follow. To answer these, we need a much better handle on the PIWI/piRNA activity in individual cells, across tissues and even up to the level of population effects in diverse environmental contexts.

Despite the intellectual draw of untangling the complexity of the piRNA biology in mosquitoes and its relevance to host-virus interactions, little is known about this pathway. The current knowledge has mostly been generated in model organisms by rigorous and elegant combinations of immunohistochemistry and next-generation sequencing methods. The availability of many antibodies has enabled the characterization of protein-protein and protein-small RNA interactions and the characterization of cellular localization of PIWI proteins. Well-annotated model genomes along with increasingly robust and affordable small RNA sequencing have been applied to identify sequences and patterns of piRNAs and piRNA clusters as well as to compare piRNA populations among experimental conditions. This body of knowledge provides a basis for studies in non-model organisms. Unfortunately, studies in non-model arthropods suffer from a paucity of specific antibodies and, while sequencing can be used in various experimental contexts, the absence of good reference genomes for several arthropods and *Aedes* species, except for *Ae. aegypti*, makes high-precision bioinformatic analyses difficult (Richards, 2018).

Excellent work in cell lines has enabled us to make headway in light of these impediments (Schnettler et al., 2013; Varjak et al., 2017; Joosten et al., 2019; Ma et al., 2020), but the expansion of mosquito PIWIs from germ-line restricted functions (**Figure 1C**) leaves us with many questions about multi-tissue functions such as might be relevant to virus regulation that are better answered in live mosquitoes (Akbari et al., 2013; Wang et al., 2018). It is therefore valuable to develop our ability to perturb the function of this pathway *in vivo*, but direct manipulation of endogenous piRNA sequences for direct hypothesis testing is not yet straight-forward in any organism. Although transgenesis and targeted genome editing using Cas9 in mosquitoes were accomplished by 1998 and 2015 respectively, opening up the possibility use genome modifications to explore the piRNA biology, the first report to do so came out this year (Coates et al., 1998; Jasinskiene et al., 1998; Basu et al., 2015; Dong et al., 2015; Kistler et al., 2015; Suzuki et al., 2020).

Similar to many processes that are involved in pathogen transmission in mosquitoes, such as host-seeking, blood-meal acquisition and digestion, and pathogen traversal of the gut, the piRNA pathway is sufficiently specialized that the closest model organism, *Drosophila melanogaster*, offers little for extrapolation to mosquitoes (Nouzova et al., 2019). This exacerbates the investigational lag induced by technological disadvantages and provides impetus to aggressively pursue new discoveries in disease vector biology. Because arthropod host stages of human disease pathogens are the most tractable targets for pathogen control, studies into specialized mosquito functions have led to novel approaches to disease control (Shaw and Catteruccia, 2019). Thus, the piRNA-pathway biology is an apt example of a pool of knowledge that, while potentially beneficial to efforts to control mosquito-borne disease, is just out of reach. We submit that a more aggressive application of genetic engineering will lengthen that reach and that existing genetic tools are not being applied to their full potential.

## The Particular Necessity of Genetic Engineering in Mosquitoes for Pirna Research

**Figure 2:**
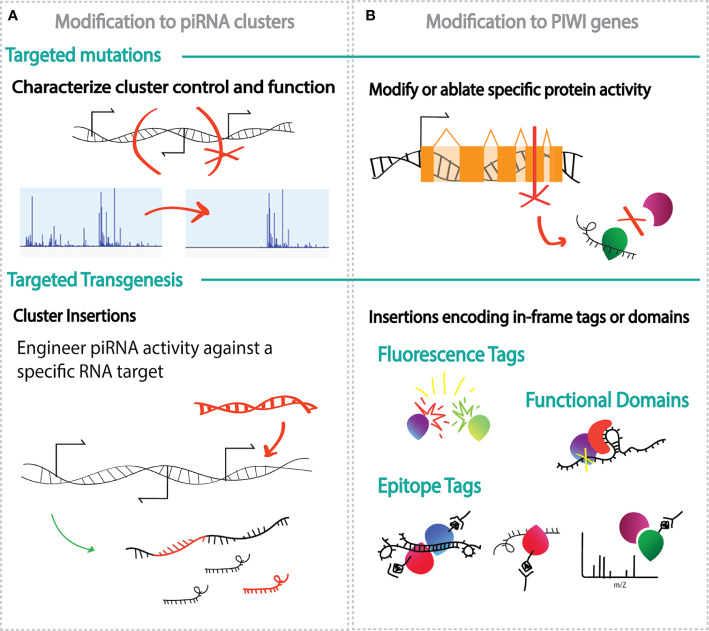
Schematic examples of genetic engineering applications to explore ethe biology of the piRNA pathway. **(A)** Modifications of the piRNA clusters through target mutations (top) and insertion of new DNA sequence (transgenesis, bottom) to identify regulatory elements within the cluster or generate piRNAs against chosen targets; **(B)** modification to the *PIWI* genes through targeted mutations (top) and/or transgenesis (bottom) and paired with methods such as fluorescence microscopy, immunoprecipitation, mass spectrometry and sequencing, to uncover in specialization of each PIWI proteins and protein partners.

We can imagine many ways in which molecular genetics, especially genetic engineering and sequencing, can allow us to answer the outstanding questions in piRNA biology (**Figure 2**). For example, direct targeting of mosquito genomes by programmable endonucleases will allow us to not only encode piRNA sequences within clusters, but also to test elements of the clusters that may be involved in expression, such as promoters, enhancers and repressors (**Figure 2A**) (Olovnikov et al., 2014). Target mutations in *PIWI* genes sequences can allow us to produce specific protein modifications to inhibit or alter function (**Figure 2B**). The scarcity of specific antibodies also can be addressed using genetic engineering; targeted transgenesis, insertion of new DNA into a gene, can be used to add fluorescence or epitope tags, such as FLAG or Myc, onto the endogenous PIWI protein. These tags can be visualized or immunoprecipitated using well characterized antibodies to characterize proteins localization or determine proteins and piRNA populations bound to a specific PIWI under different experimental conditions (**Figure 2B**) (Stadler et al., 2013; Tassetto et al., 2019). Similarly, transgenesis can be used to encode functional domains onto PIWI genes, such an RNA tether domain that can be used to detect PIWI cleavage activity on synthetic reporter transcript (Keryer-Bibens et al., 2008). Genetic manipulation can allow us to overcome the challenges of genes suppression and ablation in the germline where the piRNA pathway is greatly active, but which is mostly inaccessible to RNAi-based injection methods of knockdown and where much of the gene function is necessary for reproduction (Huvenne and Smagghe, 2010; Balakrishna Pillai et al., 2017).

## The General Impetus to Expand Genetic Engineering for Molecular Biology in Disease Vectors

In general, molecular genetic applications in mosquitoes is largely being moved forward by laboratories with defined, direct objectives for developing genetic technologies for mosquito control, but there is a real advantage in applying resources to vector biology with the explicit goal of understanding the basic biology of the vector. Technology-driven research should remain a major focus, but laboratories that are developing applications in disease control have largely driven discovery. Resources should also be put toward exploring basic biology for the sake of understanding unique cellular mechanisms of disease vectors, with the implications for disease control in mind. This will lay a strong foundation for inspiration and development of new technologies. A timely example from bacteriology is that investigations into CRISPR/Cas biology led to one of the most widespread revolutions in genetic technologies (Ishino et al., 2018). Taking advantage of the ingenuity of curious researchers to answer difficult and interesting questions, a serious commitment to basic biology will produce technological innovations.

Similarly, the more complete our fundamental understanding of mosquitoes and pathogenesis at the molecular level, the more likely our new efforts to manipulate mosquitoes and pathogens at that level for disease control will succeed. From our example of the piRNA pathway, if organisms can learn to recognize DNA in their genome as foreign (and putatively deleterious), it is possible that mosquitoes identify certain parts of transgenes as foreign and repress them (Adelman et al., 2004; Franz et al., 2009). If we understand what contributes to such recognition, we can engineer transgenic mosquitoes to avoid it. When we face an immense and long-standing problem like vector-borne disease, an earnest pursuit of lasting solutions requires efforts and resources to complete our knowledge of the basic biology of disease vectors.

We highlight the need for a concerted effort to translate genetic tools currently available in model organisms to mosquito and other disease vectors. The potential shift in momentum that we are hoping will occur will require coordination and persistence. An encouraging example of this is the creation of the field of Disease Vector Biology which was inspired by a coincidence of the resurgence of mosquito-borne diseases, a recognition that we were critically under-equipped to address this situation and a conviction that an application of modern genetic tools would lead to innovations that would bolster the resources available (Beaty et al., 2009). A collaborative and well-organized effort to translate techniques that were available for *Drosophila* genetics into mosquitoes paid off: in the early 2000s a renewed effort was made to eradicated malaria and leishmania and incredible gains have been made in decreasing the burden of disease spread caused by arthropod vectors in the decades that followed (Alonso and Noor, 2017; Benelli and Beier, 2017). Remembering the gains made in the first malaria eradication agenda and the resurgence that followed, we can reflect that the gains that *aren’t* made can reverse progress.

In light of the advancement of molecular genetic methods in model organisms, the current lag in the translation of molecular genetic methods in mosquitoes highlights major knowledge gaps in our understanding of vector biology. We propose the following areas of focus to support the robust application of molecular genetics to fill these gaps:

1. **Pursue and Support Difficult Projects for New Discovery in the Biology of Mosquitoes**. These projects are often difficult and long, but the investment is worthwhile as novel methods will be required for specialized experimentation in arthropod-borne diseases. An analogous moment in the history of the field of vector biology is the accomplishment of *Drosophila* genome transformation using the P-element (Bachmann and Knust, 2008) and subsequent efforts to translate this technology into mosquitoes. What was not widely known or demonstrated at the time was that transposable elements generally behave differently in distinct organisms and in this case the P-element is not effective for mosquito genome transformation. When this became apparent, assays to find and test the activity of alternate transposable elements led to the genome transformation in mosquitoes, 16 years after genome transformation in *D. melanogaster* (Spradling and Rubin, 1982; Coates et al., 1998; Jasinskiene et al., 1998). The lesson here is that the technologies we need will not necessarily be directly translatable from one system to another and in extension that time, energy and funding should be allocated to make necessary discoveries.
2. **Improve and Publish Information on Insect Rearing and Handling Generally, and in Relationship to Genetic Manipulation and Screening Methods**. The expansion of genetic methods to laboratories that are not specialized in mosquito genetics will be supported by development and publication of methods with low-cost and straight-forward implementation, such as those available for *Anopheles* from MR4 (Methods in Anopheles Research Manual). Several groups develop methods that work for their laboratory to efficiently manage mosquitoes with limited equipment or insect-rearing space, for example the oviplate and glytube (Costa-da-Silva et al., 2013; Ioshino et al., 2018) but many of these may not be published. This can be supported by an expansion of current information hubs to support small methods publications, foster discussion and questions that can support this effort and by introducing more descriptive methods sections in the primary literature.
3. **Expand Non-Embryo Injection Strategies for RNAi and Genetic Modification to Include More Species and Transgenesis**. The translation of Cas9-mediated gene editing using adult injections have been demonstrated to efficiently support targeted mutagenesis in several species (Chaverra-Rodriguez et al., 2018; Chaverra‐Rodriguez et al., 2020; Heu et al., 2020; Macias et al., 2020). This is supported by robust and affordable PCR and sequencing techniques (Ran et al., 2013; Bell et al., 2014; Carballar-Lejarazú et al., 2020). Expansion of this will include both translation to more vector species and engineering the techniques for DNA and RNA delivery for heritable transgenesis and RNA interference methods.
4. **Improve the Efficiency of Targeted Insertions**. In vector species in which embryo injections methods are available, rigorous testing of methods currently used in other systems to improve the efficiency of homology-directed repair will improve the manipulation of genes of interest through the introduction of a visible marker of mutation by transgenesis. A goal in mosquito species that could serve as vector model organisms, such as *Ae. aegypti* and *Anopheles stephensi*, would be to mirror the situation for *D. melanogaster* in which a transgenic line exists for most genes of interest with a variety of endogenous tags.
5. **Improve Genome Annotations and Expand Vector Sequencing**. As traditional vector control methods such as insecticides have become less effective, alternative methods involving genetic manipulation of mosquitoes are being investigated. Reliable and accurate genome assemblies of vector species and genome resequencing data of individuals from different populations and sub-species are not only essential to develop newer genetic editing approaches but also a collective tool to advance our understanding of vector species biology, gene expression, immunity and global variability.
6. **Expand Training Among Vector Biologists in Current and Emerging Genetic Methods and Use Them**. Many methods for genome investigation and manipulation are currently available for mosquitoes, but not widely used (Adelman et al., 2008; O’Brochta et al., 2012; Adelman et al., 2016; Häcker et al., 2017; Adolfi and Lycett, 2018; Reid et al., 2018). Efforts to remedy this are, for example, the course on the Biology of Disease Vectors, a technical short course on Insect Genetic Techniques and peer-to-peer training opportunities available through the IGTRCN. An important part of expanding this training may be for labs to recognize that many methods already exist for use by groups that have not traditionally used genetic techniques. For laboratories in countries directly impacted by vector-borne disease, genetic technologies can be combined with accessibility to relevant samples and colonies. These are more likely to conduct field tests with the knowledge gained and so should be a focus for training and support in genetic engineering methods. In parallel, labs globally that are already using existing techniques and that leverage genetic methods for their biological investigations, such as those studying mosquito chemo-sensation, are taking an important part in building momentum (DeGennaro et al., 2013; McMeniman et al., 2014; Matthews et al., 2019). These goals are supported by infrastructures and networks such as INFRAVEC-2 that provides coordination of resources and projects in vector-borne disease. Developing genetic engineering methods and coordinating the expansion of these methods will open the door to a phase of research that can freshly energize our efforts against vector-borne disease with new knowledge and new technologies.

With these areas of focus in mind, we can move the state of mosquito transgenic technologies forward enough to support a more robust effort to approach questions, as in those surrounding the piRNA biology in mosquitoes, that will grant us access to a molecular mechanisms currently out of reach and likely to produce insight and innovation for the control of vector-borne diseases. Doing so will provide basic knowledge and molecular methods that will feed inspiration and innovation for new approaches to vector and pathogen control and will bolster current control applications.

## Data Availability Statement

The original contributions presented in the study are included in the article/supplementary material; further inquiries can be directed to the corresponding author.

## Author Contributions

VM, UP, MB, and JR wrote the manuscript. All authors contributed to the article and approved the submitted version.

## Funding

This research was funded by a Huck Innovative and Transformative Seed Fund award to VM, by NIH grants R01AI150251, R01AI128201, and R01AI116636 to JR, by NSF grant 1645331 to JR, by USDA Hatch funds (Accession #1010032; Project #PEN04608) to JR; by a Human Frontier Science Program Research grant (RGP0007/2017) to MB, by the Italian Ministry of Education, University and Research FARE-MIUR project R1623HZAH5 to MB, by a European Research Council Consolidator Grant (ERC-CoG) under the European Union’s Horizon 2020 Programme (Grant Number ERC-CoG 682394) to MB; and by the Italian Ministry of Education, University and Research (MIUR): Dipartimenti di Eccellenza Program (2018–2022) to the Dept. of Biology and Biotechnology “L. Spallanzani,” University of Pavia.

## Conflict of Interest

The authors declare that the research was conducted in the absence of any commercial or financial relationships that could be construed as a potential conflict of interest.
